# A Compact Statistical Model of the Song Syntax in Bengalese Finch

**DOI:** 10.1371/journal.pcbi.1001108

**Published:** 2011-03-17

**Authors:** Dezhe Z. Jin, Alexay A. Kozhevnikov

**Affiliations:** Department of Physics, The Pennsylvania State University, University Park, Pennsylvania, United States of America; University College London, United Kingdom

## Abstract

Songs of many songbird species consist of variable sequences of a finite number of syllables. A common approach for characterizing the syntax of these complex syllable sequences is to use transition probabilities between the syllables. This is equivalent to the Markov model, in which each syllable is associated with one state, and the transition probabilities between the states do not depend on the state transition history. Here we analyze the song syntax in Bengalese finch. We show that the Markov model fails to capture the statistical properties of the syllable sequences. Instead, a state transition model that accurately describes the statistics of the syllable sequences includes adaptation of the self-transition probabilities when states are revisited consecutively, and allows associations of more than one state to a given syllable. Such a model does not increase the model complexity significantly. Mathematically, the model is a partially observable Markov model with adaptation (POMMA). The success of the POMMA supports the branching chain network model of how syntax is controlled within the premotor song nucleus HVC, but also suggests that adaptation and many-to-one mapping from the syllable-encoding chain networks in HVC to syllables should be included in the network model.

## Author Summary

Complex action sequences in many animals are organized according to syntactical rules that specify how individual actions are strung together. A critical problem for understanding the neural basis of action sequences is how to derive the syntax that captures the statistics of the sequences. Here we solve this problem for the songs of Bengalese finch, which consist of variable sequences of several stereotypical syllables. The Markov model is widely used for describing variable birdsongs, where each syllable is associated with one state, and the transitions between the states are stochastic and depend only on the state pairs. However, such a model fails to describe the syntax of Bengalese finch songs. We show that two modifications are needed. The first is adaptation. Syllable repetitions are common in the Bengalese finch songs. Allowing the probability of repeating a syllable to decrease with the number of repetitions leads to better fits to the observed repeat number distributions. The second is many-to-one mapping from the states to the syllables. A given syllable can be generated by more than one state. With these modifications, the model successfully describes the statistics of the observed syllable sequences.

## Introduction

Complex action sequences in animals and humans are often organized according to syntactical rules that specify how actions are strung together into sequences [1], [2]. Many examples are found in birdsong. Songs of birdsong species such as Bengalese finch [3]–[5], sedge warbler [6], nightingale [7], and willow warbler [8] consist of a finite number of stereotypical syllables (or notes) arranged in variable sequences. Quantitative analysis of the action syntax is critical for understanding the neural mechanisms of how complex sequences are generated [1], [3], [5], [9], [10], and for comparative studies of learning and cultural transmissions of sequential behaviors [11].

Pairwise transition probabilities between syllables are widely used to characterize variable birdsong sequences [3], [4], [7], [8]. This is equivalent to using the Markov model to capture the statistical properties of the syllable sequences. The Markov model is a generative statistical model of sequences, and consists of a set of states. Here the states are mathematical abstractions; they can correspond to concrete neural substrates in specific neural mechanisms of birdsong generation. There is a start state and an end state, which correspond to the start and the end of the sequences, respectively. For each syllable, there is one corresponding state. A state sequence starting from the start state and ending at the end state is produced through probabilistic transitions from one state to the next, and the corresponding syllable sequence is generated. The transition probabilities between the states depend only on the state pairs, and are set to the observed pairwise transition probabilities of the associated syllables. More sophisticated models allow chunks of fixed syllable sequences to be associated with state transitions, with a possibility that a syllable appears in different chunks [5], [12], [13]. However, no detailed statistical tests of these state transition models have been performed, and their validity as quantitative descriptions of the birdsong syntax remains unclear.

In this paper, we analyze the songs of Bengalese finch. We demonstrate that the Markov model fails to capture the statistical properties of the observed sequences, including the repeat number distributions of individual syllables, the distributions of the N-grams (sequences of length N) [14] and the probability of observing a given syllable at a given step from the start of the sequences. We introduce two modifications to the Markov model and show that the extended model is successful in describing the syntax of the Bengalese finch songs. The first modification is adaptation. Syllable repetitions are common in the Bengalese finch songs. Allowing the repeat probabilities of syllables to decrease with the number of repetitions leads to a better fit of the repeat number distributions. The second modification is many-to-one mapping from the states to the syllables. A given syllable can be generated by more than one state. Even if the transitions between the states are Markovian, the syllable statistics are not Markovian due to the multiple representations of the same syllables. The resulting model, which we call a partially observable Markov model with adaptation (POMMA), has history-dependent transition probabilities between the states and many-to-one mappings from the states to the syllables. The POMMA successfully describes the statistical properties of the observed syllable sequences. It is consistent with the branching chain network model of generating variable birdsong syntax, in which syllable-encoding chain networks of projection neurons in the premotor song nucleus HVC are connected in a branching topology [10], [15].

## Results

Spontaneous vocalizations of two Bengalese finches were recorded in an acoustic chamber using a single microphone over six (Bird 1) and five (Bird 2) days, respectively. Vocal elements (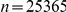, Bird 1; 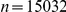, Bird 2) were isolated from the recorded pressure waves (Materials and Methods). In the following, we first present the analysis of Bird 1 and then Bird 2.

### The songs of Bird 1

**Figure 1:**
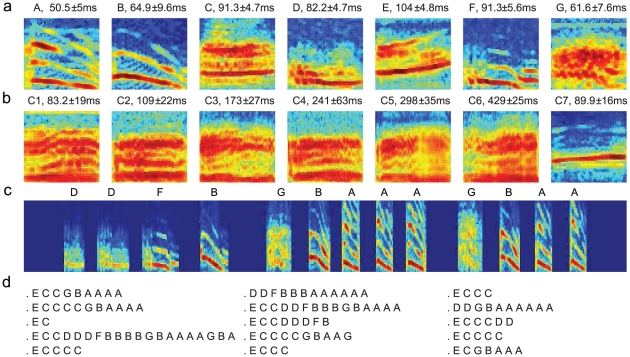
Spectrograms and song sequences (Bird 1). **a**. Spectrograms of song syllable types. **b**. Spectrograms of call types. The durations of the syllable and call types are shown on top of the spectrograms. **c**. Spectrogram of an example song. Syllable types are shown on top. The duration of the song measured from the start of the first syllable to the end of the last syllable is 1.4s. **d**. Examples of the syllable sequences. The frequency range of the spectrograms are 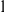-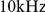.

For Bird 1, the vocal elements were clustered into 25 types according to the similarities of their spectrograms (Materials and Methods). We identified seven types of vocal elements as song syllables (Figure 1a, 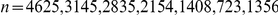 for syllables A to G, respectively). The rest were call notes (14 types; 7 examples shown in Figure 1b; C1 and C2 were the the most frequent call notes with 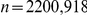, respectively) and noise. The song syllables were distinguished by rich structures in the spectrograms and tight distributions of the durations (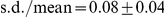), (Figure1a), and frequently appeared together in long sequences (sequence length mean 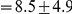) with small inter-syllable gaps (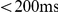) (Figure 1c–d). The gaps between the consecutive syllables were filled with silence or small noisy fluctuations; no call notes or unidentified vocal elements were in them. In contrast, the call notes had broad or simple spectra and more variable distributions of the durations (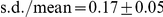), and appeared in short sequences (sequence length mean 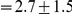). All consecutive sequences of the song syllables with inter-syllable gaps smaller than 200ms were assigned as song sequences. Additionally, syllable E (Figure 1a), which predominantly appeared at the start of the sequences obtained above, was assigned as a start syllable such that whenever syllable E appeared for the first time and was not following another E, a new song sequence was started. Thus, a long sequence containing *k* non-continuous E's in the middle was broken into *k*+1 song sequences. Altogether, we ended up with 1921 song sequences. Sequences of call notes can precede or follow song sequences, and these call notes were considered to be introductory notes.

### The Markov model

**Figure 2:**
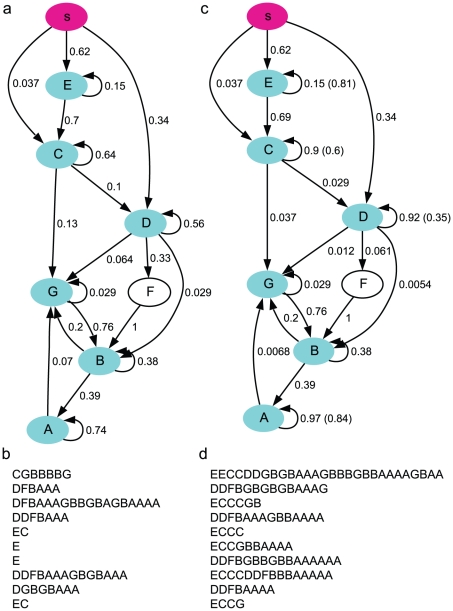
The Markov syntax of the syllable sequences (Bird 1). **a**. The Markov model. The pink oval represents the start state. The end state is not shown. The cyan ovals are the states with finite probabilities of transitioning to the end state. The numbers near the transition lines indicate the transition probabilities. **b**. Examples of syllable sequences generated from the Markov model. **c**. The Markov model with adaptation. The numbers in parenthesis are the adaptation parameter 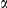. **d**. Examples of syllable sequences generated from the Markov model with adaptation.

A simple statistical model of the song sequences is the Markov model, which is completely specified by the transition probabilities between the syllables. For each syllable, there is a corresponding state; additionally, there is a start state (symbol *s*) and an end state (symbol *e*), as shown in Figure 2a. We computed the transition probability *p_ij_* for the state *S_i_* associated with syllable *i* to the state *S_j_* associated with syllable *j*, from the observed song sequences as the ratio of the frequency of the sequence *ij* over the total frequency of syllable *i*. Transitions with small probabilities (*p_ij_*<0.01) were excluded.

To evaluate how well the Markov model describes the statistics of the observed song sequences, we generated 10000 sequences from the model, and compared three statistical properties of the generated sequences and the observed sequences. The method of sequence generation is as follows. From the start state, one of three states 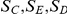 associated with syllables C, E, D can follow with probabilities 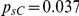, 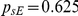, 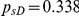, respectively (Figure 2a). A random number *r* is uniformly sampled from 0 to 1. If 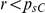, 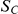 is selected (the state following the start state is 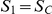), and the generated sequence starts with C. If 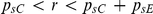, 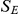 is selected (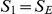), and the sequence starts with E. If 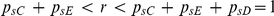, 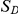 is selected (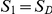), and the sequence starts with D. From the selected state 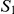, the next state 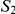 can be selected similarly according to the transition probabilities from 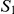. This process of sampling random numbers and selecting the next state and syllable is continued until the end state is reached, generating a specific syllable sequence. Examples of the generated syllable sequences are shown in Figure 2b.

**Figure 3:**
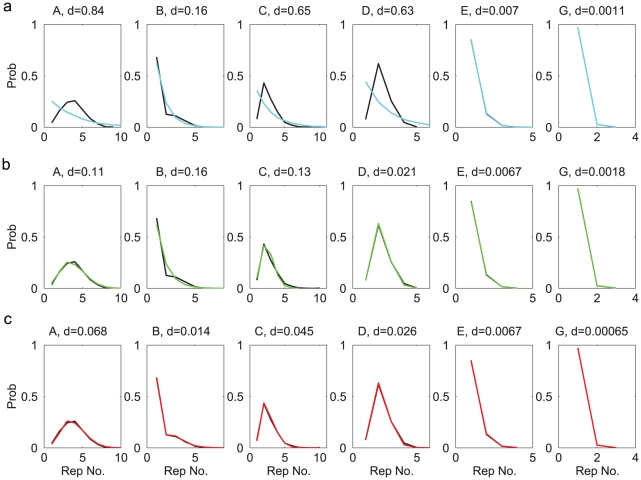
Comparisons of the repeat distributions for syllables A,B,C,D,E,G (Bird 1). The black curve in each graph is from the observed syllable sequences. **a**. Comparison to the distributions from the Markov model (cyan curves). **b**. Comparison to the distributions from the Markov model with adaptation (green curves). **c**. Comparison to the distributions from the POMMA (red curves). The differences between the model and the observed curves are indicated with the 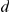-values above each graph.

The first statistical property to be compared was the distribution of the syllable repeats. Except syllable F, all syllables appeared in repetitions, and the number of repeats were variable. For each syllable, we constructed the probability distribution of the repeat numbers by counting the frequencies of observing a given number of repeats in the observed song sequences. The distributions are shown as black curves in Figure 3a. We also constructed the repeat number distributions from the sequences generated from the Markov model. These are shown as cyan curves in Figure 3a. For syllables E and G, the comparisons are favorable. However, for other syllables the distributions clearly disagree. To quantify the difference between two distributions 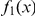 and 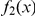, we defined the maximum normalized difference *d*, which is the maximum of the absolute differences divided by the maximum values in the two distributions, i.e. 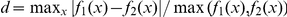. The *d*-values for syllables A, B, C, D, E are 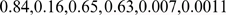, respectively. The major difference is that, for syllables A, C, D, the observed distributions peak at repeat number 4, 2, 2, respectively, while the generated distributions are decreasing functions of the repeat numbers. Indeed, if the probability of returning to state 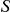 from itself is a constant 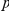, the probability of observing 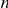 repeats of the associated syllable is 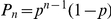, which is a decreasing function of 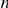. Therefore the Markov model is incapable of producing repeat number distributions having maxima at 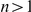.

**Figure 4:**
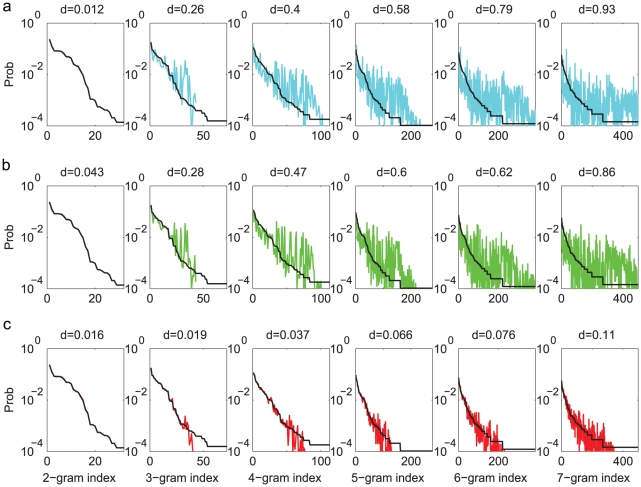
Comparisons of the N-gram distributions (Bird 1). **a**. The Markov model. **b**. The Markov model with adaptation. **c**. The POMMA. The conventions are the same as in Figure 3.

The second statistical property to be compared was the N-gram distribution. An N-gram is a fixed subsequence of length 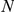. For example, syllable sequences EC and AA are 2-grams; ECC and AAA are 3-grams; etc. We constructed the probability distributions for 2- to 7-grams in the observed song sequences by counting the frequencies of a given subsequence. The results are shown in Figure 4a as black curves, with the N-grams sorted according to decreasing probabilities. We also computed the probability distributions of the corresponding N-grams in the generated sequences. The results are shown in Figure 4a as cyan curves. The distributions for 2-grams agree very well, which is expected, since the Markov model was constructed with the transition probabilities, which are equivalent to the 2-gram distributions. The distributions are quite different for 3- to 7-grams, with 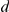-values ranging from 0.26 to 0.93 (Figure 4a).

**Figure 5:**
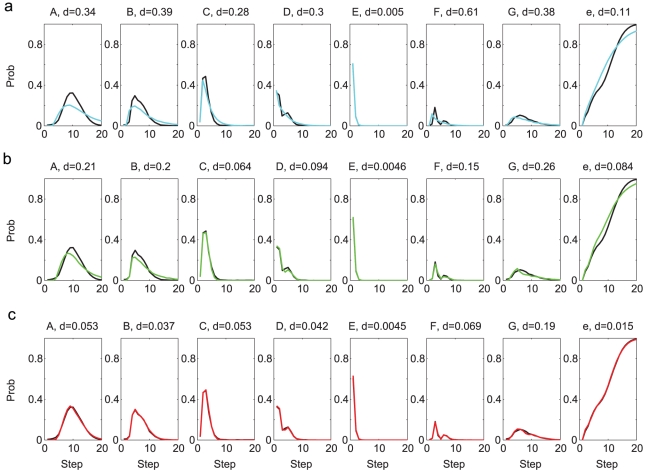
Comparisons of the probabilities of finding the syllables and the end (denoted with e) at a given step from the start (Bird 1). **a**. The Markov model. **b**. The Markov model with adaptation. **c**. The POMMA. The conventions are the same as in Figure 3.

The final statistical property to be compared was the step probability of the syllables, which is defined as the probability of observing a syllable at a given step from the start. The step probabilities for all syllables computed from the observed song sequences, as well as the step probability of the end symbol 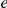, which describes the probability of observing that a sequence has ended at or before a given step, or equivalently, the cumulative distribution function of the sequence length, are plotted as black curves in Figure 5a; and those from the generated sequences are plotted as cyan curves. The comparison for syllable E is quite good (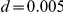). But the differences between the probabilities for other syllables and the end symbol 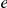 are large, as indicated by the *d*-values ranging from 0.11 to 0.61.

**Figure 6:**
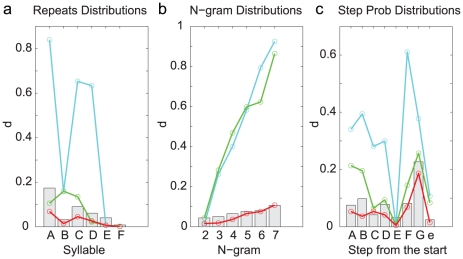
Summary of the differences between the model-generated and observed distributions (Bird 1). The 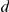-values are shown for all distributions. Cyan curves are from the Markov model, green curves from the Markov model with adaptation, and the red curves from the POMMA. The gray bars are the benchmarks obtained from the observed syllable sequences charactering the 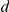-values expected due to the finite size of the samples. **a**. The repeat distributions. **b**. The N-gram distributions. **c**. The probabilities of observing syllables and the end in a given step from the song start.

Because the number of the observed song sequences is finite, even a perfect statistical model that would exactly reproduce the Bengalese finch songs cannot lead to zero *d*-values when compared to the observed distributions. One way of assessing the goodness of fits is to use benchmarks for the *d*-values created from the observed syllable sequences. We split the observed sequences into two groups by randomly assigning each sequence with a probability 0.5. One group is considered as generated by a perfect statistical model and compared against the other group. For each group we computed the repeat number distributions, the N-gram distributions, and the step probability distributions. The distributions from the two groups were compared to obtain the *d*-values. We performed the random splitting 500 times and constructed distribution profiles for each *d*-value. These profiles characterized the fluctuations of the *d*-values due to the finite number samplings of the observed sequences. For each *d*-value, we chose the 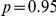 point in the profile as the benchmark. This means that the probability that the *d*-value is smaller than the benchmark is 0.95. The benchmarks are plotted as gray vertical bars in Figure 6. A good statistical model of the syllable sequences should produce *d*-values smaller than the benchmarks or close to them. The *d*-values obtained from the Markov model, plotted as the cyan curves in Figure 6, are mostly far beyond the benchmarks. It is clear that the Markov model fails to capture the statistical properties of the songs of Bird 1.

### The Markov model with adaptation

One way of extending the Markov model is to allow the transition probabilities to change depending on the state transition history. There are many possible formulations of such dependence. Adaptation, in which the transition probabilities are reduced as the state transitions are repeatedly revisited, is one formulation motivated by the observation that repeated activations of synapses and neurons reduce their efficacy [16]–[18].

Ideally, all transition probabilities should be subject to dynamical changes depending on the histories of the state transitions in the Markov model. But such a model is difficult to analyze. We therefore considered a simple model in which only the return probabilities of the states from themselves are adaptive. In particular, the return probability 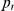 of a state is reduced to 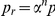 after 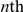 repetition of the associated syllable. The transition probabilities to all other states are mutiplied by a factor 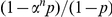 to keep the total probability normalized. Here 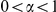 is the adaptation parameter, and 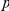 is the return probability when 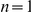. The probabilities recover to original values once the dynamics moves on to other states. In this Markov model with adaptation, the probability of observing 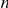 repetitions is given by 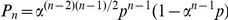 (Materials and Methods). We fitted the parameters 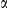 and 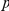 for the states with self-transitions in the Markov model (Figure 2a) using the repeat number distributions in the observed song sequences. The resulting model is shown in Figure 2b, which is identical to the Markov model (Figure 2a) except that the return probabilities for the states associated with syllables A, C, D, E are adaptive, with 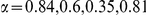, respectively. Fittings for syllables B and G did not lead to an adaptive model (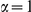), so the associated return probabilities are unchanged.

To evaluate the Markov model with adaptation, we again generated 10000 song sequences and compared the repeat number distributions, the N-gram distributions, and the step probabilities to the observed song sequences. The generation procedure was the same as in the original Markov model, except that the return probabilities were adaptive as prescribed above. The repeat number distributions, shown as green curves in Figure 3b, are much improved compared to the Markov model. In particular, the peaked distributions of syllables A, C, D are well reproduced. This demonstrates that the adaptation is capable of producing peaked repeat number distributions. Adaptation did not improve the comparisons of the N-gram distributions (Figure 4b). Adaptaion improved the comparisons of the step probabilities for syllables C, D, F but not for syllables A, B, D and the end symbol 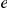 (Figure 5b). The *d*-values (green curves in Figure 6) compared to the benchmarks confirm these observations. The Markov model with adaptation is a better statistical model for song sequences of Bird 1 than the Markov model; however, it is still not capable of accurately describing all statistical properties.

### Partially Observable Markov Model With Adaptation (POMMA)

In the Markov model and its extension with adaptation, each syllable is associated with one state. Hence the number of states is equal to the number of the syllables, plus two if we count the start and end states (we will exclude the start and end states when we count the number of states in a model). However, it is possible that there is more than one state corresponding to one syllable. This many-to-one mapping from the states to the syllables enables the state transition models to describe more elaborate statistical properties of syllable sequences [10]. With the many-to-one mapping, the number of states can be larger than the number of syllables. When this is the case, some of the states are “hidden”, and cannot be simply deduced by counting the number of syllable types. This kind of model is often referred to as “partially observable Markov model” (POMM) [10], [19], and is a special case of the hidden Markov model (HMM) in which each state is associated with a single symbol. We tested whether introducing many-to-one mapping in addition to the adaptation, which leads to a “partially observable Markov model with adaptation” (POMMA), would better explain the statistical properties of the observed song sequences.

**Figure 7:**
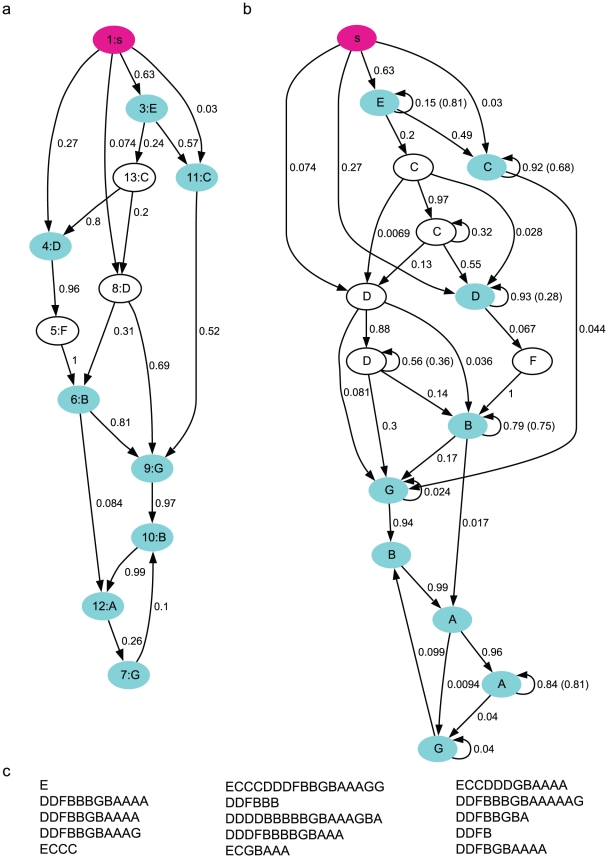
The POMMA (Bird 1). **a**. The POMM derived from the observed syllable sequences with syllable repetitions taken out. The numbers in the ovals are the state labels. **b**. The POMMA derived from the model shown in **a** with the repetitions fitted with adaptation models. **c**. Examples of syllable sequences generated from the POMMA shown in **b**. The conventions are the same as in Figure 2.

To derive a POMM from observed sequences, we developed a state merging method, in which the sequences are translated into a POMM with tree transition structure, and the states are merged if they have equivalent statistical properties and deleted if they are rarely reached (Materials and Methods). To incorporate adaptation to syllable repetitions, we first derived a POMM with the non-repeat versions of the song sequences, in which the repeats of syllables were ignored but the number of repeats were recorded. For example, the non-repeat version of a song sequence ECCDDFBBGBAA is E(1)C(2)D(2)F(1)B(2)G(1)B(1)A(2), where the repeat numbers are in the parenthesis. While creating the tree-POMM and merging the states, the repeat numbers were kept track of, so that the repeat number distribution for each state could be constructed. After following the POMM derivation procedure, there were 18 states in the model. The resulting model was evaluated by generating 10000 sequences following the state transitions from the start state. If a state with no repeat syllable was reached, the syllable associated with the state was generated. If a state with repeat syllables was reached, a repeated sequence of the syllable was generated with the repeat number sampled from the repeat number distribution associated with the state. The sequence stopped if the end state was reached. The generated sequences were compared with the observed sequences for the repeat number distributions of each syllable, the N-gram distributions, and the step probabilities of each syllable and the end symbol. We further tested deletion of each state and mergers of all pairs of states with the same syllables, while monitoring the *d*-values of the three statistical properties. The deletions and mergers were accepted if the *d*-values fell below the benchmarks or they were less than the corresponding *d*-values of the model with the 18 states. The resulting POMM, shown in Figure 7a, has 11 states. Syllables B, C, D, G are associated with two states each, and syllables A, E, F have one associated state each.

We next modeled the repeat number distributions in each state with the adaptation model described previously. For some states, the adaptation model was not adequate to fit well the repeat number distributions (cosine-similarity of the distributions 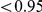 with best fitting parameters; Eq.(1) in Materials and Methods). In such a case, the state 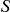 was split into two serially connected states 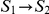. The transitions and associated probabilities to 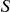 were set to 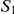, and 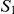 and 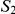 emitted to the same states and probabilities as 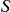. 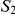 has a self-transition with probability 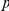 and adaptation parameter 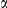, while 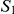 has no self-transition but has a transition probability 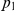 to 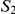. The repeat number distribution with these parameters is given by 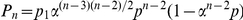 (Materials and Methods). The parameters were fit with the nonlinear least square fitting procedure. Each state-splitting thus introduced one more state and one more parameter to the model, and was adequate to fit well the observed repeat number distributions when necessary. The resulting POMMA is shown in Figure 7b. Three states associated with syllables A, C, D were split. Altogether, there are 14 states, and the number of states for syllables A to G are 2, 2, 3, 3, 1, 1, 2, respectively.

We generated 10000 syllable sequences from the POMMA (examples shown in Figure 7c), and compared with the observed song sequences the repeat number distributions (Figure 3c), the N-gram distributions (Figure 4c), and the step probabilities (Figure 5c). The comparisons are excellent. All *d*-values fall below or close to the benchmarks, as shown with the red curves in Figure 6. In contrast, the *d*-values for the Markov model are mostly far beyond the benchmarks, as shown with the cyan curves in Figure 6. The *d*-values for the Markov model with adaptation are also larger than those for the POMMA, as shown with the green curves in Figure 6. In particular, the *d*-values for the N-gram distributions are far beyond the benchmarks and the *d*-values of the POMMA. Thus, the POMMA is a much better model than the Markov model or the Markov model with adaptation.

### The songs of Bird 2

**Figure 8:**
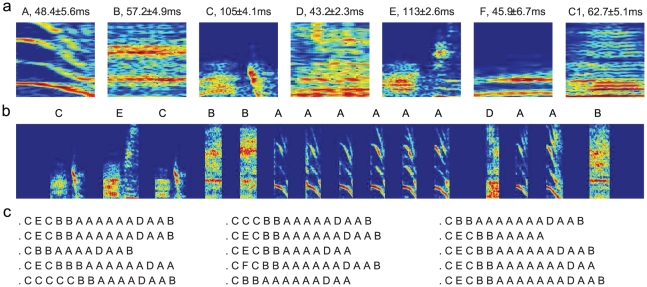
The syllables and song sequences (Bird 2). The convention is the same as in Figure 1.

We repeated the analysis for songs of Bird 2. The vocal elements were clustered into 7 types, with 6 types identified as song syllables (Figure 8a, 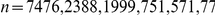 for syllables A to F, respectively) and one type identified as the introductory note (Figure 8a, C1, 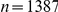). The song sequences occurred in long sequences (mean length 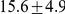 s.d.), with the gaps between consecutive syllables smaller than 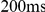. The introductory note appeared with repeats preceding the song sequences, and had much smaller volume compared to the song syllables. Less call notes were recorded for Bird 2 than for Bird 1 since the song sequences could be distinguished from the calls based on the lengths of the consecutive sequences of vocal elements with the gaps 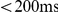. A total of 845 song sequences were used for deriving the models.

### Comparisons of the three models

**Figure 9:**
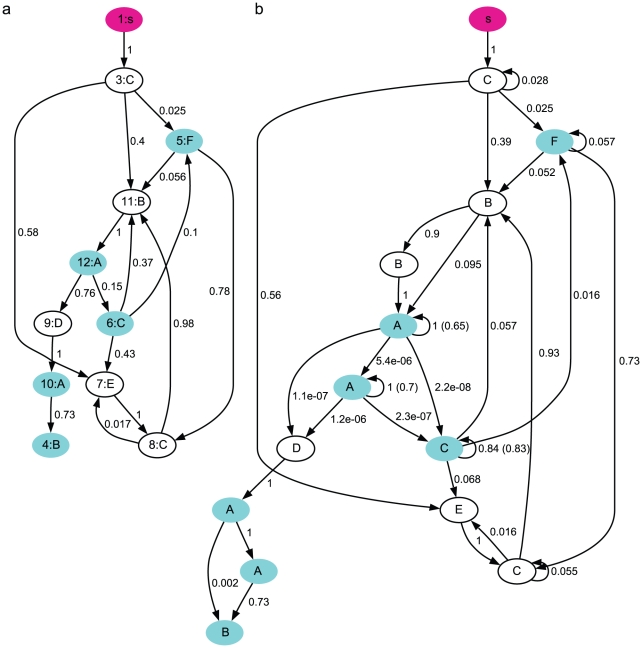
The POMMA (Bird 2). **a**. The POMM derived from the observed syllable sequences with syllable repetitions taken out. The numbers in the ovals are the state labels. **b**. The POMMA derived from the model shown in **a** with the repetitions fitted with adaptation models. The convention is the same as in Figure 2.

We derived the POMMA for Bird 2 using the same procedure as for Bird 1. The POMM derived with the non-repeat versions of the song sequences has 10 states (Figure 9a). There are two states associated with syllable A, three states with syllable C, and one state with all other syllables. The states in the POMM with syllable repeats were replaced with states with adaptive self-transition probabilities and additional states when necessary to derive the POMMA (Figure 9b). Syllable A is associated with state 12 and state 10 of the POMM. In state 12, the number of repetitions of syllable A ranges from 2 to 16 and the repetition distribution peaks at 6. We modeled this distribution by replacing state 12 with two serially connected states 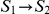, each with adaptive self-transitions (Materials and Methods). The self-transition probabilities and the adaptation parameters are 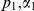 for 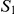, and 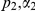 for 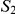. The transition probability from 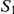 to 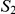 is 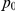. The inward transitions to state 12 of the POMM were set to 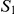 with the probabilities intact. The outward transitions from state 12 were transferred to 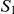 and 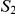, with the transition probabilities scaled to make sure that total transition probabilities out from 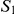 and 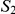 were normalized including the self-transitions and the transitions from 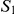 to 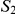. The resulting repeat number distribution with these parameters was fitted with the observed distribution using the nonlinear least square procedure (Materials and Methods), and the cosine-similarity of the fitted and the observed distributions reached 0.98. We tested simpler models of the repeat number distribution for state 12, including one state with adaptive self-transition probability and two serial states with only one state with adaptive self-transition probability, but they did not work as well. In state 10 of the POMM, syllable A repeats twice more than 99.7% of the time, with the rest being single repeats. We modeled this by replacing state 10 with two serial states 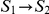 with no self-transitions, and with a small probability of not transitioning from 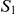 to 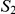 to account for the rare case of single syllable A. The inward transitions to state 10 were transferred to 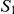, and the outward transitions from state 10 were transferred to 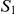 and 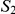, similarly as for the case of state 12. The situation is similar for syllable B in state 11, which predominantly has two repeats (90%). State 11 was replaced with two serial states with no self-transitions. The number of repetitions for syllable C in state 6 ranged from 1 to 6 and peaked at 3. This repetition number distribution was model with one state with adaptive self-transition probability. All other states with more than one repeat were accurately modeled by adding self-transitions as in the Markov model. The cosine-similarities of the fitted and the actual repeat number distributions were all greater than 0.95. The resulting POMMA, shown in Figure 9b, has 13 states (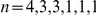 for syllables A to F, respectively).

**Figure 10:**
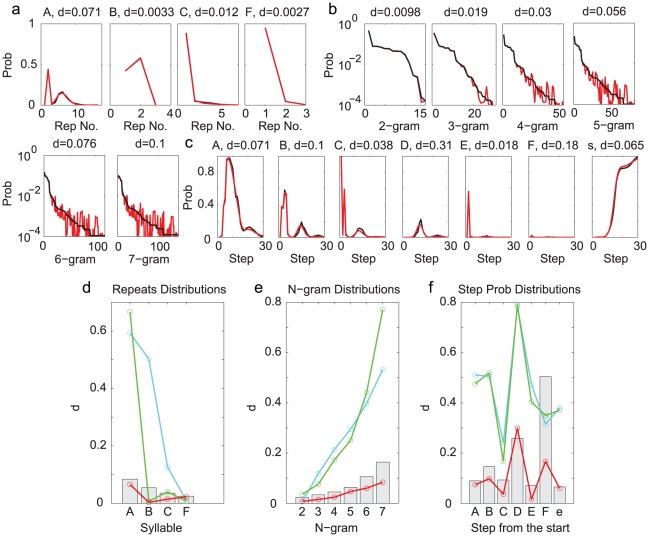
Comparisons of the models and data (Bird 2). The repeat (**a**), the N-gram (**b**) and the step probability (**c**) distributions are compared for the observed (black curves) and the POMMA-generated (red curves) sequences. **d, e, f.** Summary of the differences between the model-generated and observed distributions for the Markov model (cyan curves), the Markov model with adaptation (green curves), and the POMMA (red curves). The gray bars are the benchmarks obtained from the observed syllable sequences.

The POMMA accurately describes the statistical properties of the syllable sequences of Bird 2. We generated 10000 song sequences using the POMMA, and compared to the observed sequences the repeat number distributions, the N-gram distributions, and the step probability distributions. The comparisons are excellent (Figure 10a–c). The 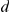-values between the model and the observed distributions are below or very close to the benchmarks obtained from the observed sequences as in the case of Bird 1 (Figure 10d–f, red curves). In contrast, the Markov model and the Markov model with adaptation, derived and evaluated following the same procedure as for Bird 1, fail to describe the statistical properties of the observed sequences (Figure 10d–f, cyan and green curves). The Markov model with adaptation cannot accurately model the repeat number distribution of syllable A, which has double peaks as shown in the first graph in Figure 10a, even though the model can accurately describe the repeat number distributions of other syllables. This contributed significantly to the inaccuracy of the Markov model with adaptation in the N-gram distributions and the step probability distributions.

### Evidence of many-to-one mapping

**Figure 11:**
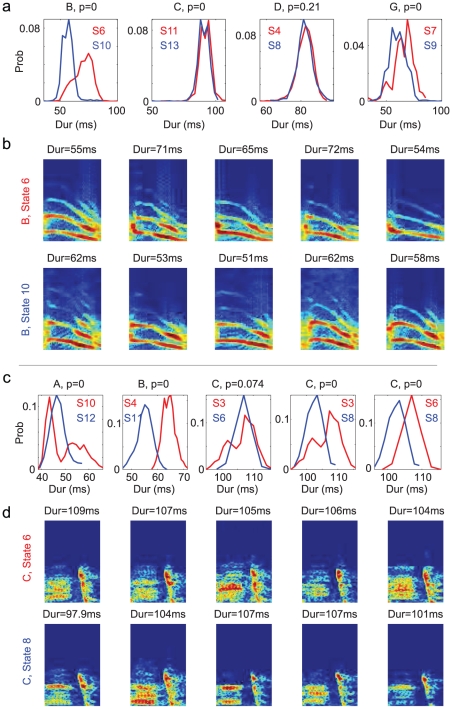
Evidence of many-to-one mapping from the states to the syllables. Panels **a** and **b** are for Bird 1, and **c** and **d** for Bird 2. **a** and **c**. Syllable durations of the same syllable types associated with different states in the POMM shown in Figure 7a and in Figure 9a, respectively. In each graph, red and blue curves are from different states. The state labels are shown with corresponding colors. The 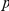-values on top test the significance that the differences of the means of the two distributions are non-zero. The syllable types are shown on top. **b** and **d**. Spectrograms of randomly selected examples of syllables of the same type associated with different states in the POMM. Durations of the syllables are shown on top. Frequency range of the spectrograms is from 1–10kHz.

In the POMM, different states can be associated with the same syllable type. One possible piece of evidence of such many-to-one mapping from states to syllables can be the subtle differences that might exist in the instances of the same syllable associated with different states. For Bird 1, there are two states for syllables B,C,D,G in the POMM shown in Figure 7a. We compared the duration distributions of the same syllable types associated with different states, as shown in Figure 11a. The distributions are clearly distinctive for syllables B, C, G (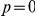, shuffle test of the significance that the difference of the means of the two distributions is none-zero; the null-distribution of the difference of the means was generated using 500 pairs of randomly shuffled distributions, and the 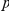-value is the two-tailed probability of the difference of the means greater than the observed value given the null-distribution). There is no clear evidence of distinctions for syllable D (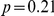). Despite the significant differences in the durations for syllables B in the two states, the spectrograms of the syllables in the two states are very similar, as shown in Figure 11b. The same is true for other syllables.

**Figure 12:**
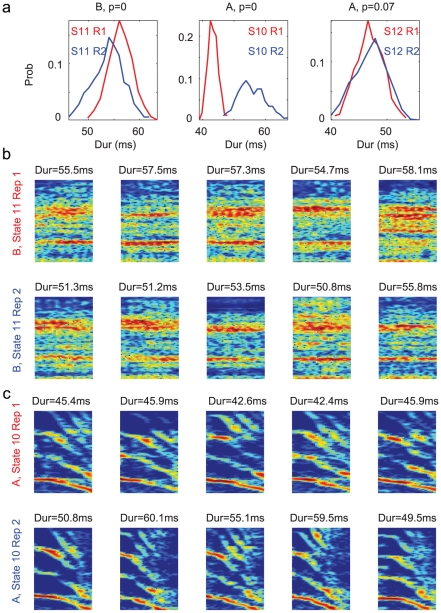
Evidence of different states representing the repeating syllables. **a.** The duration distributions of the syllables occurring the first (red curves) and the second (blue curves) in the repetitions are compared for syllable B associated with state 11 (left), syllable A associated with state 10 (middle), and syllable A associated with state 12 (right) in the POMM of Bird 2 shown in in Figure 9a. The 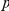-values on top test the significance that the differences of the means of the two distributions are non-zero. **b.** Spectrograms of randomly selected examples of syllables B occurring the first (top) and the second (bottom) in the repetition in state 11. **c.** Same as in **b** but for syllable A in state 10. Durations of the syllables are shown on top. Frequency range of the spectrograms is from 1–10kHz.

For Bird 2, the duration distributions of the same syllable types associated with different states are mostly distinctive (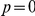 in three cases and 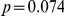 in one case), as shown in Figure 11c, while spectrally the syllables are very similar (examples shown in Figure 11d). Most interestingly, durations of the syllables associated with the same state in the POMM can also be distinctive depending on the positions of the syllables in the repetition. In Figure 12a we show three cases. The first is syllable B associated with state 11 in the POMM. The durations of syllable B in the first position of repetition is significantly longer than in the second position of the repetition (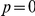). The second is syllable A associated with state 10. The durations of syllable B in the first position of repetition is clearly shorter than those in the second position (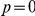). Spectrally, these sets of syllables are indistinguishable (Figure 12b for syllable B and 12c for syllable A). Both states were replaced with two serial states in the POMMA. Weaker evidence (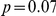) also exists for syllable A associated with state 12 in the POMM (Figure 12a), which is replaced with two serial states both with adaptive self-transition probabilities in the POMMA. The systematic variations of syllable durations on the positions in repetition supports the idea of using multiple states to model repeat number distributions associated with single states in the POMM.

Taken together, the results on syllable durations provide some evidence for the validity of the many-to-one mapping from the states to the syllables.

## Discussion

Bengalese finch songs consist of variable sequences of a finite number of syllables. We have shown that the statistical properties of the sequences are well captured by a state transition model, the POMMA, in which the repeat probabilities of the syllables adapt and many-to-one mappings from the states to the syllables are allowed. The Markov model, which has been commonly used in studies of characterizing variable birdsong sequences, is clearly inadequate for the Bengalese finch songs. The POMMA is an extension of the Markov model. As in the Markov model, each state is associated with a single syllable, and the state transitions are characterized by the transition probabilities. However, unlike the Markov model, many states are allowed to be associated with the same syllable, and the state transition probabilities can vary depending on the history of the state transitions dynamics. These extensions are motivated by considerations of the neural mechanisms of birdsong generation.

The premotor nucleus HVC (used as a proper name) is a critical area in songbird brain for song production [20]. Firing of HVC neurons that project to RA (the robust nucleus of the arcopallium) drives singing [21], [22]. Experimental evidence suggests that a syllable is produced by the bursts of spikes propagating in a chain network of HVC projection neurons [22]–[25]. A set of HVC projection neurons reliably drive the RA neurons [22], which in turn drive downstream motor neurons to produce sound. Such a chain network in HVC could be a neural representation of a single state in POMMA. Thus, the association of a state to a single syllable is a reflection of the reliability of a chain network driving the production of a syllable.

The connections from HVC to RA are learned [26]–[29]. This makes it possible that different sets of HVC projection neurons are set up during learning to drive acoustically similar syllables. In zebra finch, different neural activity in HVC has been observed during vocalizations of acoustically similar syllables [21], [30], supporting the possibility of multiple sets of HVC neurons driving the same syllable. Such a possibility of many-to-one associations from the neural sets in HVC to syllables motivates introduction of many states corresponding to one syllable in the POMMA. It is conceivable that the same syllable driven by different sets of HVC neurons have subtle differences in the acoustic features due to imperfections of learning. Indeed, we found that instances of the same syllable associated with different states in the POMMA can have significantly different duration distributions (Figure 11 and Figure 12). A recent study has shown that the acoustic features of Bengalese finch syllables can shift systematically depending on the sequences around the syllables [31], which is in agreement with our observation. There can be alternative explanations to our observations that do not require separate sets of HVC neurons to encode the same syllable. One possibility is that the sequence-dependent differences in the acoustic features are due to the history dependence of the activations of the unique set of HVC neurons driving the syllable. Another possibility is that the differences are due to the inertia of the motor periphery rather than the variations in neural activity [31]. Finally, the differences can be due to sequence dependent activations of neurons in other areas in the song system, such as RA [31]. More direct experiments, such as single unit recordings in HVC of singing Bengalese finch, are required to test unambiguously whether the many-to-one mapping from HVC to RA exits.

The POMMA can be directly mapped onto the branched chain network model of the Bengalese finch song syntax [10]. Each state of the POMMA corresponds to a syllable-encoding chain network of HVC projection neurons, and each transition 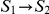 in the POMMA corresponds to the connection from the end of the synaptic chain corresponding to 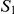 to the start of the synaptic chain corresponding to 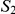. The POMMA and the network model thus have identical branching connection patterns. In the network model, spike propagation along a chain drives the production of a syllable. At a branching point, spike propagation continues along one of the connected chain networks with a probability that depends on a winner-take-all competition and noise [10], [15]. The success of the POMMA in capturing the statistical properties of the Bengalese song sequences supports the branched chain network model of Bengalese finch song syntax. A critical prediction for the network model is that, for some syllables, HVC projection neurons should burst intermittently, bursting during some instances of the syllables but not in others. This is markedly different from the case of zebra finch, in which HVC projection neurons burst reliably for each production of the song sequence [22], [25]. The prediction can be tested with electrophysiological experiments.

Adaptations are widely observed in neural systems. Continuous firing can reduce neuron excitability [18], and excitatory synapses can be less effective when activated repeatedly [16], [17]. In zebra finch, consecutive singing increases the durations of the song syllables [32]. It is possible that the slow-down of the song tempo is due to some adaptive processes in HVC. In the branched chain network model of the Bengalese song syntax, weakening connection strength from one chain network to another at a branching point reduces the transition probabilities between them [10]. These observations suggest that the transition probabilities might not be fixed. Introducing adaptive processes in the neural excitability and synaptic efficacy should lead to adaptive transition probabilities in the branched chain network model, especially for the repeated activations of a chain network, which correspond to the reduction of the self-transition probability. It remains to be seen experimentally whether HVC projection neurons or the excitatory synapses between them have the adaptive properties. It might be also possible to see the signatures of adaptation by analyzing the burst intervals of HVC projection neurons during syllable repetitions, or the burst intervals of RA neurons. The observation that burst intervals in RA neurons steadily increase with song sequence repetition in zebra finch [32] suggests that similar effect could be observed in Bengalese finch.

We emphasize that adaptation is important for reducing the complexity of the state transition model. It is possible to include syllable repetitions in the POMM, with no adaptations of the transition probabilities, and accurately describe the statistical properties of the Bengalese finch songs (Materials and Methods; supplementary Figures S2–S4). However, compared to the POMMA with adaptation, the number of states is larger. While the POMMA has 14 and 13 states for Bird 1 and Bird 2 (Figures 7b and 9b), respectively, the POMM has 20 and 18 states (Figures S2 and S3). In the POMM, many states are needed to produce the peaked repeat number distributions such as that of syllable A in Bird 2 (Figure 10a). The difference of the number of states in the POMM and the POMMA should increase with the number of syllables with peaked repeat number distributions. It is the significant reduction of the model complexity that motivates our choice of the model with adaptation (the POMMA) rather than the non-adapting model (the POMM).

We have used multiplicative reduction of the repeat probabilities. It remains to be investigated whether other formulations of the adaptation can be similarly or even more effective. In our approach, only the repeat probabilities are adapted. A more consistent model should allow adaptation and recovery in all transition probabilities, such that the state transition dynamics depends on the history of the entire syllable sequence, not just the syllable repetitions. This approach might be important if there are repeats of short sequences such as ABABABAB, in which the transition probabilities from A to B and B to A might need to be adapted. But such a model is difficult to derive from the observed sequences. In our data, repetitions of short sequences were rarely seen, hence adapting only the repeat probabilities of single syllables was adequate. We have shown that adaptation alone is not sufficient to augment the ability of the Markov model to describe the Bengalese finch songs, and the many-to-one mapping from the states to the syllables is necessary. However, we cannot rule out the possibility that the more consistent model with all transition probabilities adaptive, and perhaps with more complex forms of adaptation, can eliminate the requirement for the many-to-one mapping.

The POMMA is closely related the hidden Markov model (HMM) [33], which is widely used to model sequential structures in human languages [14], [33], [34] and genomes [35], [36]. In the HMM, the transitions between the states are as in the Markov model, but each state is allowed to emit all symbols (or syllables in birdsong case) with some probability dependent on the state. The flexibility of the state and the symbol associations makes the HMM much more capable of capturing statistical properties of sequences than the Markov model. To apply the HMM to birdsong, however, it makes more sense to require that a state can be associated with a single syllable only, if the correspondence between the model and the neural dynamics of birdsong generation is considered [10]. HVC neurons reliably activate RA neurons [22], and there is no evidence that activation of the same sets of HVC or RA neurons can probabilistically produce multiple syllables. The HMM with the restriction that one state emits one symbol is the POMM [10], [19]. The POMM is distinguished from the Markov model in that a syllable can be associated with multiple states (many-to-one mapping from the states to the syllables). Even though the transitions between the states are Markovian, the syllable statistics can be non-Markovian due to the multiple representations of the same syllables [10]. The HMM with no one-to-one restriction does not lead to a more compact model than the POMMA for the Bengalese finch songs (Materials and Methods). To achieve the level of the accuracy of the POMMA, the HMM needs close to 18 states for both Bird 1 and Bird 2 (Figures S7), which is similar to the POMM. Indeed, most states in the HMMs predominantly emit one syllable (Figures S5 and S6), and the structures of the HMMs and the POMMs are similar for both birds.

There are previous efforts of describing Bengalese finch song sequences with state transition models [12], [13]. Chunks of syllable sequences, which are fixed sequences of syllables, were extracted from the observed sequences and used as the basic units of the state transition models [12], [13]. A syllable can appear in many chunks, hence these models implicitly contain the many-to-one mapping from the states to the syllables. But the chunk extractions and the state models were not derived from the statistics of the observed sequences. Furthermore, the models were not tested against the observed song sequences for statistical properties. In contrast, the POMMAs were derived from and tested with the observed song sequences.

Although there is a close connection between the POMMA and the branched chain network model of how HVC generates variable syllable sequences in Bengalese finch [10], [15], the POMMA or the POMM can be compatible with alternative neural mechanisms, including feedback control of sequences through RA to HVC projections [31], syntax generation in other nuclei upstream to HVC or RA in the song system [12], [37], [38], noisy recurrent networks in HVC [39], and branched chain networks of inhibitory HVC interneurons [40]. It is also possible that different statistical models can be derived from these mechanisms. More detailed analyses of the alternative mechanisms are needed to see whether they can produce syllable sequences with statistics compatible to the observed Bengalese finch songs.

There should be a family of equivalent POMMAs for the songs of a Bengalese finch. For example, the same repeat distributions can always be modeled with more states. The POMMA that we have derived is the simplest model that is consistent with the data. Given this insight, we expect that the neural representation of the syntax should be similar to the derived POMMA but most likely not identical. We have developed a state merging method for deriving the POMM from the observed syllable sequences. It is possible to use the well-established methods of training the HMM [33] to derive the POMM. We observe that our method is faster than the training methods of the HMM. A more detailed analysis of the state merging method is needed to quantify its speed and convergence properties.

In conclusion, we have derived a compact POMMA that successfully describes the statistical properties of Bengalese finch songs. Our approach can be useful for modeling other sequential behaviors in animals and statistical properties of sequences in general.

## Materials and Methods

### Vocalization recording

Acoustic recordings were performed with a boundary microphone (Audio-Technica PRO44). Microphone signals were amplified and filtered (8th-order Bessel high-pass filter with 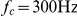 and 8th-order Bessel low-pass filter with 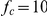
kHz, Frequency Devices). The filtered signals were digitized with a 16-bit A/D converter (PCI-6251, National Instruments) with a sampling rate of 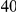
kHz.

### Vocal elements and spectrograms

Vocal elements were defined as continuous sounds bounded by silent periods. Thresholding the amplitudes of the pressure waves is a common approach of isolating vocal elements in birdsongs [31], [41], [42]. We developed a similar method. From the pressure wave 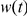 of a vocalization, the oscillation amplitude 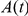 at time 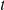 was obtained by finding the maximum of 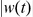 in the interval of one oscillation cycle that contains 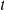. The amplitude was further transformed to 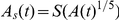, where 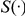 is a smoothing function that uses the second order Savitzky-Golay filter with 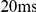 window (801 data points). Vocal elements were isolated by detecting continuous regions in 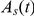 that were above a threshold function 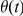. The threshold function was obtained in 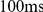 moving windows (step size 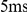); in each window, the threshold was set at the 0.3 point from the floor 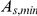 of 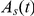 to the local maximum of 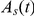 in the window. The floor 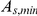 is the characteristic value of 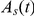 in the regimes with no sound, and was identified as the position of the lowest peak in the histogram of the values of 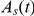 for all 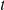. A detected region was excluded if the total area above 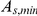 was smaller than 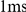 multiplied by the difference between the maximum value 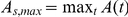 and 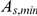; or if the maximum value of 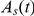 in the region minus 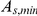 was smaller than 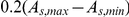; or if the width of the region was less than 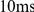. These exclusions ensured that most noisy fluctuations were not counted as vocal elements. The results of the vocal element isolations were manually checked and adjusted by plotting out the waveforms in conjunction with the boundaries of the vocal elements to ensure that no obvious mistakes were made. The parameters used in the above procedure were empirically determined to yield the best results in our dataset. They should be adjusted if the procedure is used for other recordings of birdsong.

The waveform of an isolated vocal element was transformed into a spectrogram 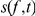, which is the energy density at frequency 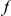 and time 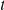. The frequency was restricted to 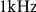 to 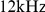. The spectrogram was computed with the multi-taper method [43] (time-bandwidth product, 1.5; number of tapers, 2) with 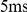 window size and 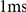 step size (software from http://chronux.org). The frequency was discretized into grids with 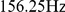 between adjacent points. To exclude silent periods at the beginning and the end of the vocal element, the time span of the spectrogram was redefined to the region in which the total power in the spectrum at each time point exceeded 5% of the maximum of the total powers.

### Types of vocal elements

We used a semi-automated procedure to cluster the vocal elements into separate categories. Similarities between the vocal elements were defined and used in a clustering algorithm. The final results were visually inspected and adjusted by plotting the spectrograms of all vocal elements in the clusters.

The similarity between the vocal elements was defined as follows. The spectrogram 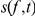 was considered as a sequence of spectra at the discrete time points. The spectrum at each time point was smoothed over the frequency domain using the second order Savitzky-Golay filter with window size of 5 frequency points. The smoothed spectrum was further decomposed into a slowly varying background 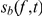 by smoothing with the second order Savitzky-Golay filter with window size of 20 frequency points; and peaks 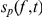 by subtracting out 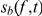. The relative importance of the peaks compared to the background was characterized by the weight 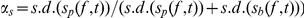, where 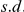 is the standard deviation of the distribution over the frequency domain.

The spectrum at 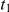 of 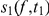 was compared to the spectrum at 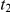 of 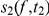 by computing 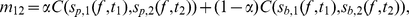
which is the weighted sum of the cosine-similarities between the peaks and between the backgrounds. Here 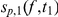 and 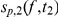 are the peaks and 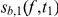 and 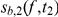 are the backgrounds of 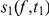 and 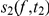, respectively. The cosine-similarity 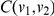 of two vectors (or distributions) 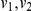 was defined as 
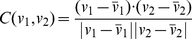
(1)where 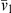 and 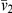 are the means and 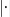 is the norm. 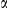 is the maximum of the weights across all time points of the two syllables. If 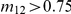, the two spectra 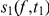 and 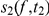 were considered the same (denoted 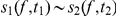). Otherwise the two spectra were defined as distinctive.

The similarity between two syllables was characterized by the longest common subsequence (LCS) between them. A common subsequence was defined by a set of time points 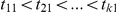 in syllable 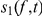 and a set 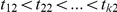 in syllable 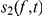, such that the spectra at corresponding time points are the same, i.e. 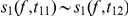, 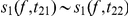, ..., 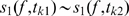. There was an additional restriction that corresponding time points did not differ by more than 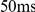, i.e. 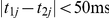 for all 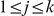. The length of the common subsequence is 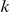. LCS is the common subsequence with the maximum length. A long LCS indicates that the two syllables are similar, while a short LCS indicates they are dissimilar. We defined the similarity score of two syllables as the length of LCS divided by the mean of the lengths of the two syllables.

Types of vocal elements were identified by clustering 4000 vocal elements using a core-clustering algorithm, modified from the algorithm described in Jin et al [44]. The algorithm is based on the distance between vocal elements, defined as one minus the similarity score, and consists of the following steps. (1) For each vocal element, find the list of nearby vocal elements with distances less than 0.1. (2) Among the vocal elements that are not yet part of a cluster, select the one with at least 5 nearby vocal elements and the smallest mean distances to its nearby vocal elements as the core point of a new cluster. (3) Assign all unclustered vocal elements that are in the nearby-list of the core point to the new cluster. All vocal elements that are in the nearby-list but already clustered are reassigned to the new cluster if their distances to the core points of their respective clusters are larger than their distances to the new core point. (4) Repeat steps (2–3) until no new cluster could be created. (5) Merge clusters. Two clusters are merged if at least 5% of the vocal elements in each cluster had small distances (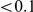) to the vocal elements in the other cluster. (6) Assign vocal elements that are not yet clustered. A vocal element is assigned to the cluster that had the maximum number of members whose distances to the vocal element are less than 0.15. In some cases, individual clusters contained separate vocal element types that had subtle differences but distinguishable. Such clusters are split into new clusters.

Once the types of vocal elements were identified with the clustering algorithm, we used the following procedure to classify all vocal elements that were not already clustered. (1) Identify the center of each cluster as the vocal element that has the minimum mean distances to all other vocal elements in the cluster. (2) Compute the distances from the vocal element to be assigned to the cluster centers. The three clusters with the lowest distances are selected. (3) Compare the durations of the vocal elements in the selected clusters to the duration of the candidate vocal element, and select 20 (or less if the cluster size is smaller than 20) from each selected cluster that are closest. (4) Compute the distances from the candidate vocal element to the selected vocal elements. (5) Assign the vocal element to the cluster to which the most of the selected vocal elements with the distances smaller than 0.2 belong. (6) If none of the selected vocal elements have distances less than 0.2, do not assign the candidate vocal element. The unclustered vocal elements were grouped into 2000 blocks, and their mutual distances were computed. The clustering and identifying procedures were repeated until no more clusters emerge. During this process, clusters were merged if they were subjectively judged as similar by inspecting the spectrograms and the mutual distances between the members of the clusters. Individual vocal elements were reassigned to different clusters if necessary.

The final results of the clustering of the vocal elements were validated and adjusted by visual inspections of the spectrograms.

### Repeats number distributions with adaptation

In the case of a state with self-transition, the transition probability is 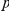 initially but is reduced to 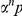 after 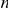 repetitions of the state, where 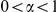 is the adaptation parameter. The probability of having 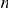 repeats is then 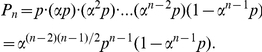



More complex repeat distributions can be modeled with more states. One model has two serial states 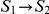. Both are associated with the same syllable, and only 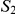 has self-transition. The transition probability from 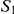 to 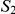 is 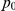, and the self-transition probability of 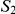 is 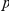 initially but undergoes adaptation with the adaptation parameter 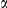. The probability of observing one repeat is given by 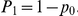



The probability of observing 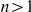 repeats is given by 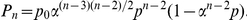



Another model with two serial states allows both 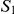 and 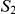 to have self-transitions with parameters 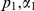 for 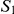 and 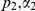 for 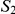. The probability of transitioning to 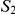 after leaving 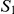 is 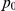. The probability of observing one repeat is 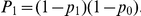



The probability of observing two repeats is 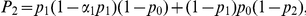
in which the first and the second terms are the probabilities of the state sequences 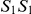 and 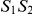, respectively. Similarly, for 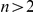, the probability of observing 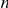 repeats is given by 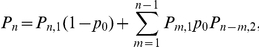
where 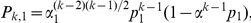
and 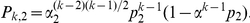



Here 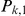 and 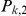 are the probabilities of repeating 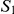 and 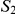 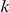 times, respectively.

The cases above were all we needed to model the Bengalese finch songs in this study. More complex models with more states can be necessary for other Bengalese finch songs, and the repeat number distributions can be similarly derived.

### Derivation of the POMM

We used a state-merging method to derive the POMM from the observed syllable sequences. The process is illustrated with an example in Figure S1 with a simple case of two syllables 1 and 2. From 5000 observed sequences (Figure S1a), a tree Markov model is constructed (Figure S1b). For each sequence, the tree model contains a unique path of state transitions from the start state. This is achieved by starting with the start state 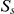 and the end state 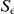 only, and adding new states as needed by finding the paths for the sequences. For example, consider the first sequence 12. At this point no states are emitted from the start state. A new state 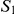 with syllable 1 is added and connected from the start state; a new state 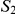 with syllable 2 is added and connected from 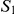; finally, 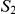 connects to the end state. With the additions of the two states, the sequence is mapped to a state transition path 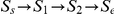. Now consider the second sequence 121. State transitions 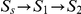 generate the first two syllables in the sequence. To generate the last 1, a new state 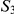 with syllable 1 is added, and is connected from 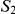 and also to the end state. Now 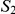 branches into 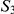 and 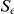. This process continues, until all observed sequences are uniqued mapped into the paths in the tree model. The transition probabilities from a state to all connected states are computed from the frequencies of the transitions observed in the sequences. The tree model is a simple POMM that is a direct translation of the observed sequences; it contains all observed sequences. However, the tree model is incapable of generating novel sequences that are statistically consistent with the observed sequences. Moreover, since each transition probability can be considered as a parameter, the number of parameters in the tree model is enormous, severely restricting its predictive power. To reduce the number of parameters, a more concise POMM is derived by merging the equivalent states in the tree model. If two states are associated with the same syllable, and the probability distributions of subsequent sequences of length 15 or smaller are similar (cosine-similarity 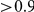), the two states are merged. This is done until no further mergers are possible. Finally, state transitions with probabilities smaller than 0.01 are eliminated, and all states that are reached less than 0.005 times in all observed sequences are also eliminated. These merging and pruning procedures lead to a concise POMM with five states for the simple example, as shown in Figure S1c. There are two states for syllable 1, which is an example of the many-to-one mapping. Indeed, the observed sequences in Figure S1c was generated with a POMM with structure identical to the one in Figure S1c and with equal transition probabilities to all connected states from a given state. The example demonstrates that the state merging method can lead to a concise POMM from observed sequences. The procedure was used to derive the POMMs for Bird 1 and Bird 2 using the non-repeat versions of the syllable sequences and keeping track of the number of syllable repetitions in each state, as described in the main text. The accuracy of the POMM from the state merging procedure was tested by generating 10000 sequences (see the main text for the generation procedure) and comparing with the observed sequences the repeat number distributions, the N-gram distributions, and the step probability distributions. The 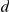-values were computed and compared with the benchmarks derived from the observed syllable sequences as discussed in the main text. The number of states in the POMM was further reduced by testing mergers of all states associated with the same syllables and testing deletions of all states. The mergers and deletions were accepted if the 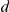-values of the resulting POMM fell below the benchmarks or they were smaller than the 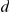-values of the original POMM. The state merging and subsequent reduction of the number of states was fully automated. The POMM derived from the above procedure were morphed into the POMMA by replacing each state associated to repeating syllables with one or more states with adaptive self-transition probabilities. Various adaptive models for the repeat number distributions were tested as described in the main text. The process of morphing the POMM to the POMMA was not automated.

To derive the POMM from the syllable sequences but include the syllable repetitions without introducing adaptation, each state associated with repeating syllables in the POMM derived with the non-repeat versions was replaced by its own POMM. The replacing POMM was derived from the repeat sequences of the syllable using the HMM training method described below. In this case, since there is only single syllable in the repeat sequences, the HMM is equivalent to the POMM. We increased the number of states in the replacing POMM until the repeat number distribution of the syllable could be reproduced with the cosine-similarity 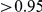. The in and out transitions in the POMM from the non-repeat versions were retained in the replacements. The resulting POMMs for Bird 1 and Bird 2 are shown in Figures S2 and S3. Direct applications of the state merging procedure did not lead to concise POMMs using the syllables sequences with repetitions. The main reason was that the syllable repetitions, especially when the mean repetition number was larger, required more sequences than available to accurately judge the statistical equivalence of the states for merging in the tree POMM.

### Derivation of the HMM

We used the Baum-Welch algorithm for training the HMM from the observed sequences [33]. A number of states is chosen for the HMM. There is a start state and an end state, which only emit the start and the end symbols, respectively. All other states can be associated with any of the syllables with the emission probabilities. The transitions from the start state to the end state and from all states to the start state were excluded. All transition and emission probabilities were set randomly initially, and adjusted with the observed sequences using the Baum-Welch algorithm until they converged (errors of the probabilities below 0.001). To avoid local minima in deriving the HMM, we repeated the training process 20 times, and selected the HMM with the maximum log-likelihood for the observed sequences. The derived HMM was evaluated by generating 10000 sequences and comparing the statistics with the observed sequences. The generation method is the same as in the Markov model, except that at each state, the syllable generated is determined from the emission probabilities at that state. The number of states in the HMM was systematically varied. The results for Bird 1 and Bird 2 are shown in Figures S5–S7.

## Supporting Information

Figure S1 An example of deriving the POMM from observed sequences. a. The observed sequences generated by a POMM with three states, two states with symbol 1 and one state with symbol 2. b. The tree-POMM derived from 5000 observed sequences. c. The derived POMM after merging equivalent states in the tree-POMM. The original model used to generate the sequences shown in a are recovered. The diagram conventions are the same as in Fig. 2.
(0.29 MB EPS)

Figure S2 The POMM for Bird 1. The POMM is derived with the syllable repetitions included. The conventions are the same as in Fig. 2.
(0.29 MB EPS)

Figure S3 The POMM for Bird 2. The POMM is derived with the syllable repetitions included. The conventions are the same as in Fig. 2.
(0.30 MB EPS)

Figure S4 Summary of the differences between sequences generated with the POMM and the observed sequences for the repeat (left), the N-gram (middle) and the step probability (right) distributions. a. Bird 1. b. Bird 2. The POMM's are shown in Fig. S2 and S3. The gray bars are the bench marks.
(0.33 MB EPS)

Figure S5 The hidden Markov model (Bird 1). The HMM with 18 states are shown. In each state, the syllable with the maximum emission probability is shown, along with the maximum emission probability. Other conventions are the same as in Fig. 2.
(0.30 MB EPS)

Figure S6 The hidden Markov model (Bird 2). The HMM with 18 states are shown. In each state, the syllable with the maximum emission probability is shown, along with the maximum probability. Other conventions are the same as in Fig. 2.
(0.27 MB EPS)

Figure S7 Summary of the differences between sequences generated with the hidden Markov models and the observed sequences for the repeat (left), the N-gram (middle) and the step probability (right) distributions. Number of states in the models are indicated with the colors: cyan, 8; green 13; red, 18; and black, 23. a. Bird 1. b. Bird 2.
(0.38 MB EPS)

## References

[1] Lashley KS, Jeffress LA (1951). The problem of serial order in behavior.. Cerebral Mechanisms in Behavior (the Hixon Symposium).

[2] Colonnese M, Stallman E, Berridge K (1996). Ontogeny of action syntax in altricial and precocial rodents: grooming sequences of rat and guinea pig pups.. Behaviour.

[3] Woolley SM, Rubel EW (1997). Bengalese finches lonchura striata domestica depend upon auditory feedback for the maintenance of adult song.. J Neurosci.

[4] Honda E, Okanoya K (1999). Acoustical and syntactical comparisons between songs of the white-backed munia (lonchura striata) and its domesticated strain, the bengalese finch (lonchura striata var. domestica).. Zool Sci.

[5] Okanoya K (2004). The bengalese finch: a window on the behavioral neurobiology of birdsong syntax.. Ann N Y Acad Sci.

[6] Catchpole C (1976). Temporal and sequential organisation of song in the sedge warbler (Acrocephalus schoenobaenus).. Behaviour.

[7] Todt D, Hultsch H (1998). How songbirds deal with large amounts of serial information: retrieval rules suggest a hierarchical song memory.. Biol Cybern.

[8] Gil D, Slater P (2000). Song organisation and singing patterns of the willow warbler, Phylloscopus trochilus.. Behaviour.

[9] Sakata J, Brainard M (2006). Real-time contributions of auditory feedback to avian vocal motor control.. J Neurosci.

[10] Jin D (2009). Generating variable birdsong syllable sequences with branching chain networks in avian premotor nucleus HVC.. Phys Rev E.

[11] Slater P (1989). Bird song learning: causes and consequences.. Ethol Ecol Evol.

[12] Hosino T, Okanoya K (2000). Lesion of a higher-order song nucleus disrupts phrase level complexity in bengalese finches.. Neuroreport.

[13] Kakishita Y, Sasahara K, Nishino T, Takahasi M, Okanoya K, Randall M, Abbass H, Wiles J (2007). Pattern Extraction Improves Automata-Based Syntax Analysis in Songbirds.. Progress in artificial life.

[14] Jurafsky D, Martin JH (2000). Speech and Language Processing..

[15] Chang W, Jin D (2009). Spike propagation in driven chain networks with dominant global inhibition.. Phys Rev E.

[16] Markram H, Tsodyks M (1996). Redistribution of synaptic efficacy between neocortical pyramidal neurons.. Nature.

[17] Abbott L, Varela J, Sen K, Nelson S (1997). Synaptic depression and cortical gain control.. Science.

[18] Sanchez-Vives M, Nowak L, McCormick D (2000). Cellular mechanisms of long-lasting adaptation in visual cortical neurons in vitro.. J Neurosci.

[19] Callut J, Dupont P, Paliouras G, Sakakibara Y (2004). A Markovian approach to the induction of regular string distributions.. Grammatical Inference: Algorithms and Applications.

[20] Nottebohm F, Stokes TM, Leonard CM (1976). Central control of song in the canary, serinus canarius.. J Comp Neurol.

[21] Yu AC, Margoliash D (1996). Temporal hierarchical control of singing in birds.. Science.

[22] Hahnloser RH, Kozhevnikov AA, Fee MS (2002). An ultra-sparse code underlies the generation of neural sequences in a songbird.. Nature.

[23] Jin DZ, Ramazanoglu FM, Seung HS (2007). Intrinsic bursting enhances the robustness of a neural network model of sequence generation by avian brain area hvc.. J Comput Neurosci.

[24] Long M, Fee M (2008). Using temperature to analyse temporal dynamics in the songbird motor pathway.. Nature.

[25] Long M, Jin D, Fee M (2010). Support for a synaptic chain model of sequence generation from intracellular recordings in the singing bird.. Nature.

[26] Herrmann K, Arnold A (1991). The development of afferent projections to the robust archistriatal nucleus in male zebra finches: a quantitative electron microscopic study.. J Neurosci.

[27] Doya K, Sejnowski T, Tesauro G, Touretzky D, TK L (1995). A novel reinforcement model of birdsong vocalization learning.. Advances in neural information processing systems. Volume 7.

[28] Fee MS, Kozhevnikov AA, Hahnloser RH (2004). Neural mechanisms of vocal sequence generation in the songbird.. Ann N Y Acad Sci.

[29] Fiete I, Hahnloser R, Fee M, Seung H (2004). Temporal sparseness of the premotor drive is important for rapid learning in a neural network model of birdsong.. J Neurophysiol.

[30] McCasland JS (1987). Neuronal control of bird song production.. J Neurosci.

[31] Wohlgemuth M, Sober S, Brainard M (2010). Linked control of syllable sequence and phonology in birdsong.. J Neurosci.

[32] Chi Z, Margoliash D (2001). Temporal precision and temporal drift in brain and behavior of zebra finch song.. Neuron.

[33] Rabiner L (1989). A tutorial on hidden Markov models and selected applications in speech recognition.. Proc IEEE.

[34] Kupiec J (1992). Robust part-of-speech tagging using a hidden Markov model.. Comput Speech Lang.

[35] Krogh A, Larsson B, Von Heijne G, Sonnhammer E (2001). Predicting transmembrane protein topology with a hidden markov model: application to complete genomes.. J Mol Biol.

[36] Durbin R, Eddy S, Krogh A, Mitchison G (2002). Biological sequence analysis..

[37] Scharff C, Nottebohm F (1991). A comparative study of the behavioral deficits following lesions of various parts of the zebra finch song system: implications for vocal learning.. J Neurosci.

[38] Olveczky B, Andalman A, Fee M (2005). Vocal experimentation in the juvenile songbird requires a basal ganglia circuit.. PLoS Biol.

[39] Yamashita Y, Takahasi M, Okumura T, Ikebuchi M, Yamada H (2008). Developmental learning of complex syntactical song in the Bengalese finch: A neural network model.. Neural Net.

[40] Katahira K, Okanoya K, Okada M (2007). A neural network model for generating complex birdsong syntax.. Biol Cybern.

[41] Janata P (2001). Quantitative assessment of vocal development in the zebra finch using self-organizing neural networks.. J Acoust Soc Am.

[42] Du P, Troyer T (2006). A segmentation algorithm for zebra finch song at the note level.. Neurocomputing.

[43] Mitra P, Bokil H (2008). Observed brain dynamics..

[44] Jin D, Fujii N, Graybiel A (2009). Neural representation of time in cortico-basal ganglia circuits.. Proc Natl Acad Sci USA.

